# Supplementary material for: O-GlcNAcylation Is Required for the Survival of Cerebellar Purkinje Cells by Inhibiting ROS Generation
Source: Antioxidants (Basel). 2023 Mar 26;12(4):806. doi: 10.3390/antiox12040806 (PMC10135177; doi:10.3390/antiox12040806)
Supplement: Supplementary file 1 [file antioxidants-12-00806-s001.zip › antioxidants-2247145-supplementary.pdf]

Supplementary Information for

O-GlcNAcylation is Required for the Survival of Cerebellar Purkinje Cells by Inhibiting ROS Generation

Fengjiao Liu [1,†], Shen Li [1,†], Xin Zhao [1,†], Saisai Xue [1], Hao Li [1], Guochao Yang [1], Ying Li [1], Yan Wu [1], Lingling Zhu [1,2], Liping Chen [1,*] and Haitao Wu [1,2,3,*]

1 Beijing Institute of Basic Medical Sciences, Beijing 100850, China
2 Key Laboratory of Neuroregeneration, Co-innovation Center of Neuroregeneration, Nantong University, Nantong 226019, China
3 Chinese Institute for Brain Research, Beijing 102206, China
* Correspondence: 2006443033@163.com (L.C.); wuht@bmi.ac.cn (H.W.); Tel.: +86-10-66931363 (H.W.)
† These authors contributed equally to this work.

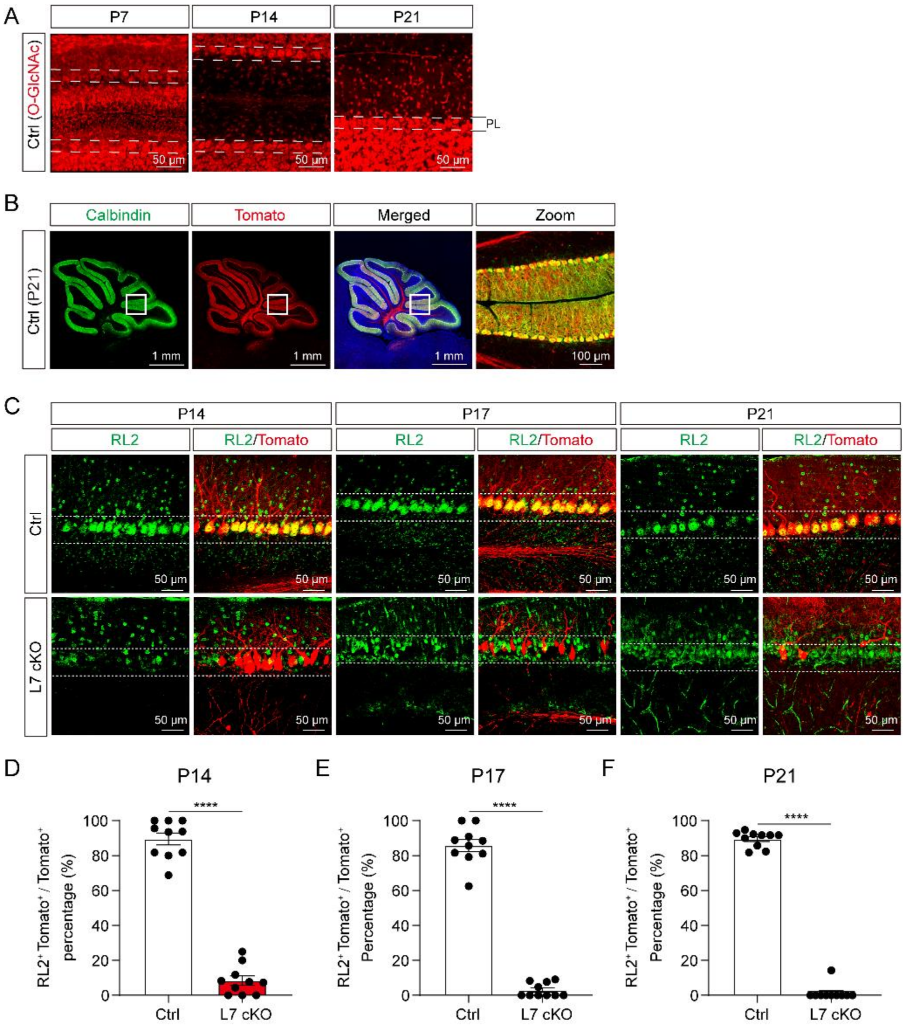

Supplementary Figure S1: Significantly decreased O-GlcNAcylation levels in PCs in L7 cKO mice compared to Ctrl littermates. (A) Immunofluorescent staining with anti-RL2 antibodies shows the high level of O-GlcNAcylation in PC layer (PL) in Ctrl mice at P7, P14 and P21, respectively (scale bar = 50 μm). (B) Ai9 reporter mice reveals that L7-Cre is specifically expressed in the PCs of cerebellum (scale bar = 1 mm or 100 μm). (C) Immunofluorescent staining with anti-RL2 antibodies shows the significantly decreased level of O-GlcNAcylation in PCs in cKO mice compared to WT littermates (scale bar = 50 μm). (D-F) Quantitative analysis of the O-GlcNAcylation in PC cells in mice at P14 (D), P17 (E) and P21(F), respectively, (mean ± SEM; ****$p < 0.0001$, n = 10).

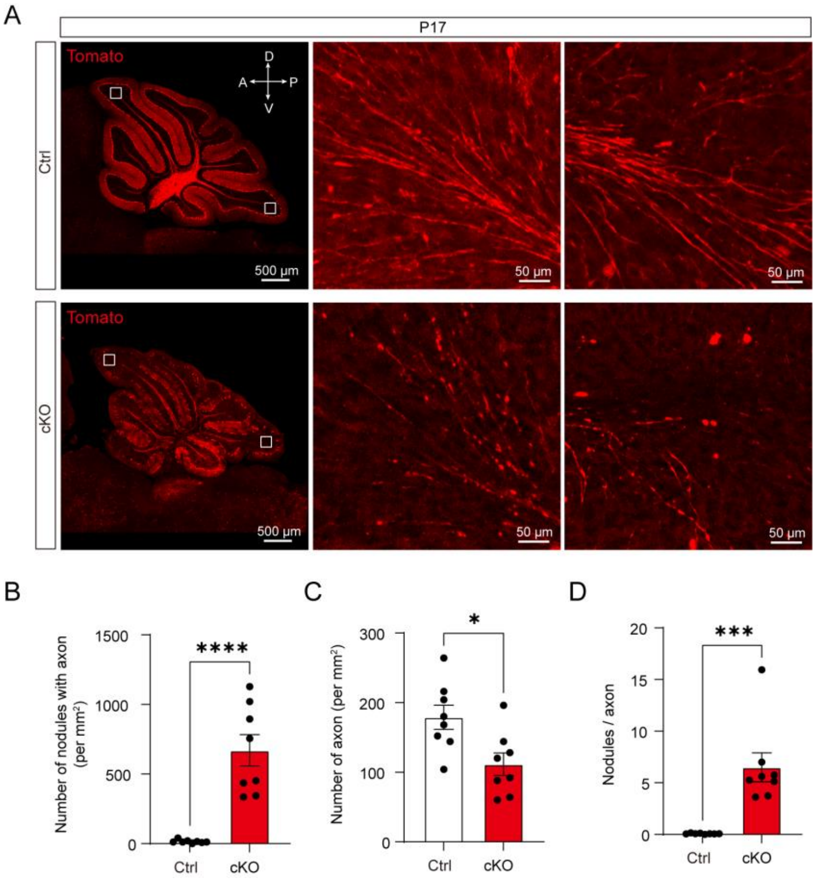

Supplementary Figure S2: Loss of O-GlcNAcylation in PCs leads to discontinuous intumescence in axons. (A) Immunofluorescent image of Tomato (red) to reveal the axon morphological changes of PCs in the cerebellar vermis of Ctrl and cKO mice (left: scale bar = 500 μm, right: scale bar = 50 μm). (B-D) Quantitative analysis of the axon morphological changes of PCs (B), number of nodules above axon (C), number of axon (D), The ratio of nodules to axon (mean ± SEM; *p < 0.05, ***p < 0.01, ****p < 0.0001, n = 8).

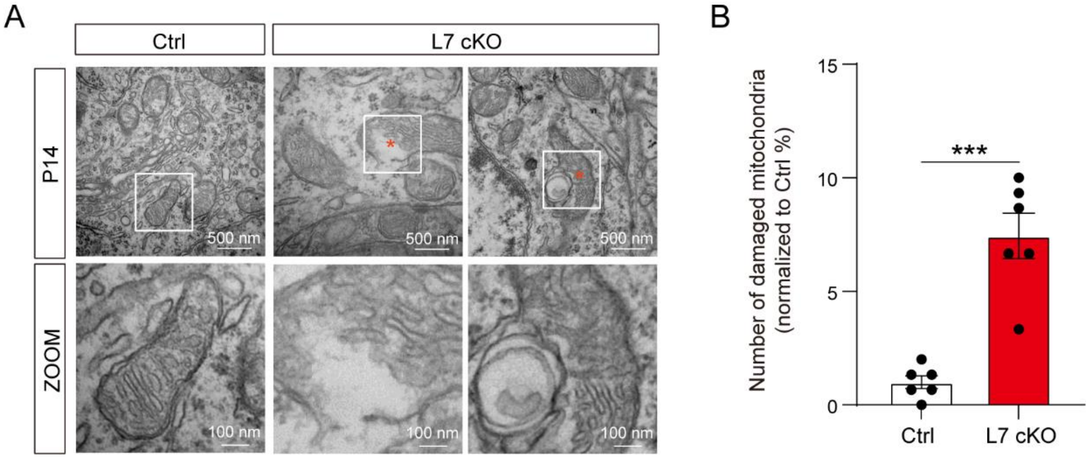

Supplementary Figure S3: Loss of O-GlcNAcylation in PCs leads to mitochondrial damage. (A) Electron micrograph of the mitochondria in PCs from Ctrl and cKO mice at P14 (scale bar = 500 nm, 100 nm). (B) Quantitative analysis of the damaged mitochondria in the PCs from Ctrl and cKO mice at P14 (mean ± SEM; ***p < 0.001, n = 6).

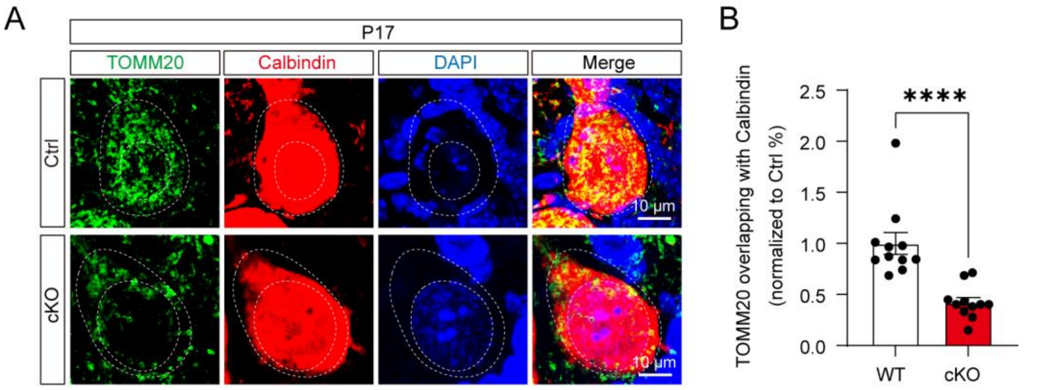

Supplementary Figure S4: Loss of O-GlcNAcylation in PCs leads to downregulation of TOMM20 expression in cKO mice. (A) Immunofluorescent staining with anti-TOMM20 and anti-Calbindin antibodies (scale bar = 10 μm). (B) Quantitative analysis of the number of TOMM20 overlapping with calbindin (mean ± SEM; ****p < 0.0001, n = 11).

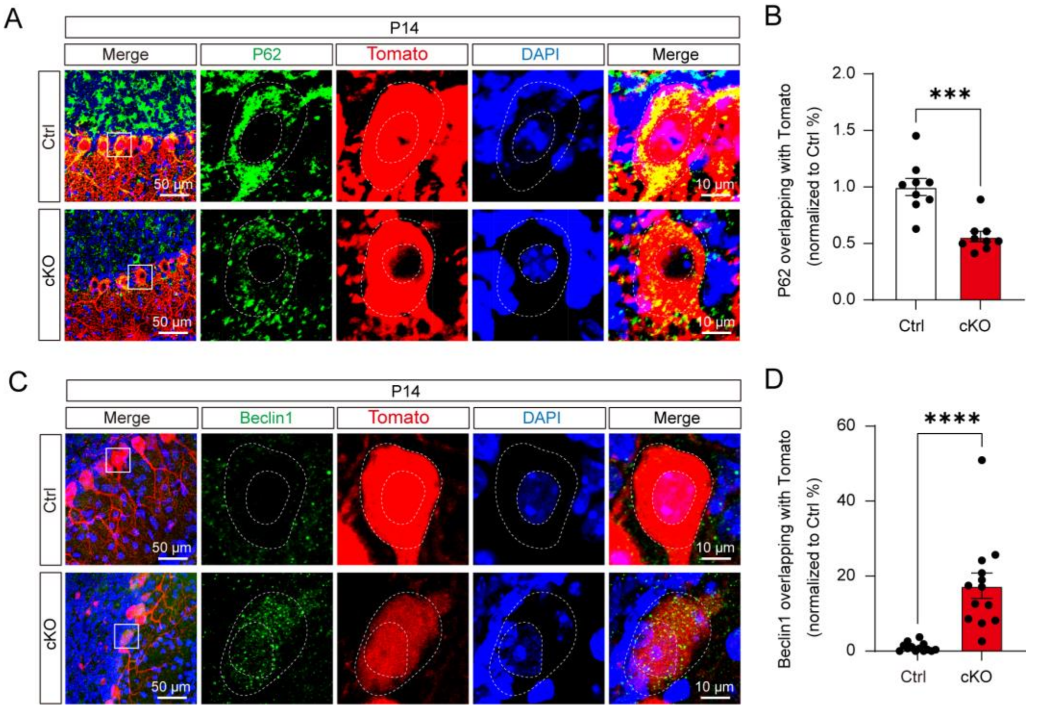

Supplementary Figure S5: Loss of OGT in PCs significantly decrease P62 protein level and increase Beclin1. (A) Co-immunofluorescent staining with anti-P62 (green) and Tomato (red) show the significantly decreased P62 expression (left: scale bar: 50 μm, right: scale bar: 10 μm). (B) Quantification of the P62 fluorescence particle of PCs in Ctrl and cKO mice at P14 (mean ± SEM; ****p < 0.0001, n = 9). (C) Immunofluorescence analysis of cellular localization of Beclin1 (green) and Tomato (red) scale bar: 50 μm). (D) Quantification of Beclin1 puncta numbers in Ctrl and cKO mice at P14 (mean ± SEM; ****p < 0.0001, n = 13).

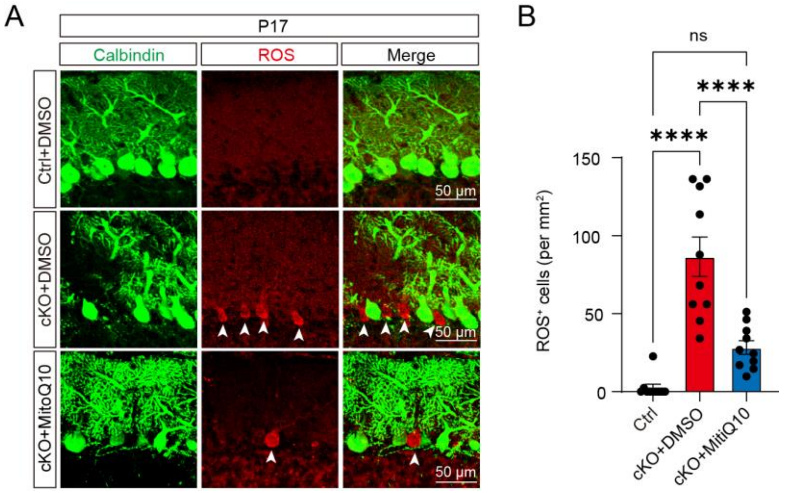

Supplementary Figure S6: Treatment of MitiQ10 significantly reduces the expression of ROS in cKO mice. (A) Immunofluorescent image of Calbindin (green) and ROS (red) in the cerebellar vermis of Ctrl and cKO mice treated with DMSO, or the cKO mice treated with MitoQ10. (Scale bar: 50 µm). (B) Quantification of the ROS+ cells in Ctrl and cKO mice treated with DMSO, or the cKO mice treated with MitoQ10 (mean ± SEM; ****p < 0.0001, n = 10).

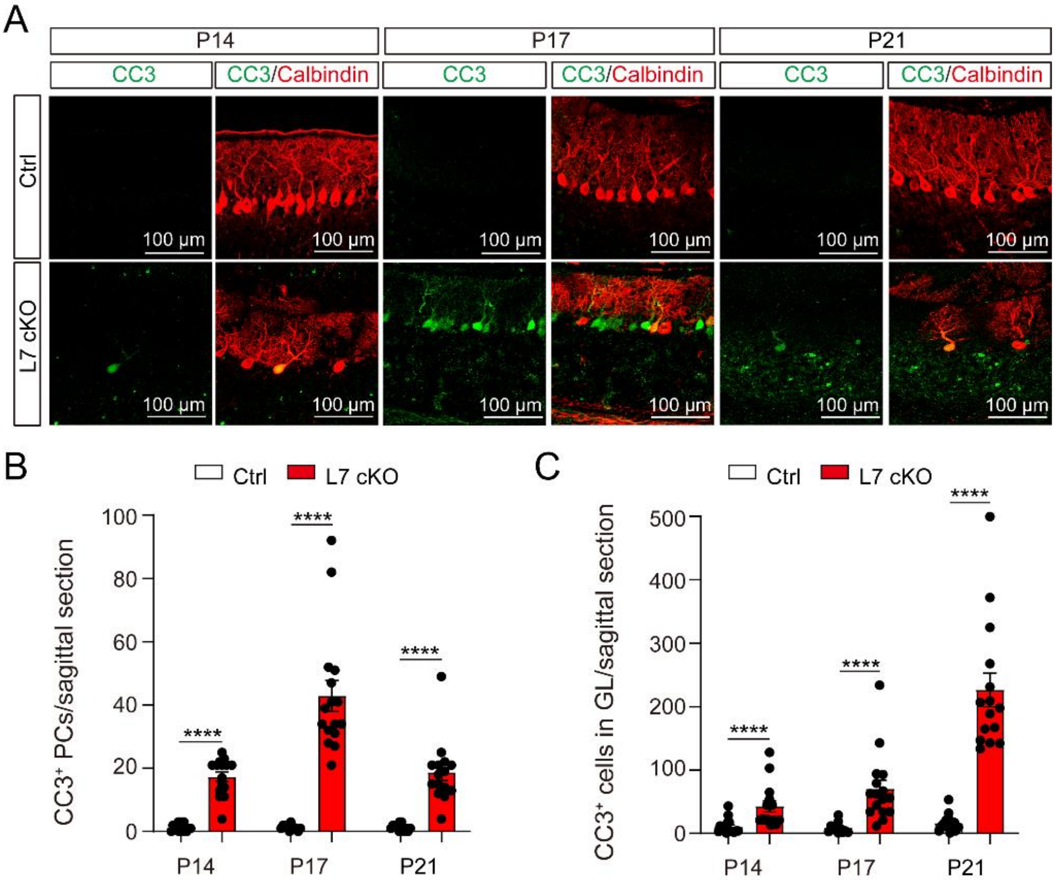

Supplementary Figure S7: Loss of O-GlcNAcylation in PCs leads to apoptosis in cKO mice. (A) Immunofluorescent staining with anti-cleaved caspase3 and anti-calbindin antibodies shows apoptosis in PCs and inner granule layer in cKO mice (scale bar = 100 µm). (B and C) Quantitative analysis of the cleaved caspase3 positive PCs (B) and cells in inner granule layer (GL) (C) per sagittal section in Ctrl and cKO mice (mean ± SEM; ****p < 0.0001, n > 10).

Supplementary Table S1. Primers used for genotyping and qRT-PCR

Primers	Sequences	gene accession number
Genotyping: L7-Cre-F	ATTCTCGTGGAACTGGATGG	NC_005856.1
Genotyping: L7-Cre-R	GGACAGGTAATGGTTGTCTGG	
Genotyping: OGT loxP-F	TCTCTCCAGCCCCACAAACTG	NC_000086. 1
Genotyping: OGT loxP-R	GACGAAGCAGGAGGGGA-GAGCAC	
Genotyping: LC3-GFP-F1	TCCTGCTGGAGTTCGTGACCG	NC_000068. 8
Genotyping: LC3-GFP-R1	TGAGCGAGCTCAAGA-TAATCAGGT	
Genotyping: LC3-GFP-F2	TTGCGAATTCTCAGCCGTCTTC ATCTCTCTCGC	NC_000068.8
Genotyping: LC3-GFP-R2	GTTAGCATTGAGCTGCAA-GCGCCGTCT	
Genotyping: Ai9-F	GGCATTAAAG CAGCGTATCC	AY678269.1
Genotyping: Ai9-R	CTGTTCCTGT ACGGCATGG	
qRT-PCR: Atg3-F	ACACGGTGAAGGGAAAGGC	NM_026402.3
qRT-PCR: Atg3-R	TGGTG-GACTAAGTGATCTCCAG	
qRT-PCR: Atg4a-F	GCTGGTATGGATTCTGGGGAA	NM_001379682
qRT-PCR: Atg4a-R	TGGGTTGTTCTTTTTGTCTCTCC	
qRT-PCR: Atg4b-F	TATGATACTCTCCGGTTT-GCTGA	NM_001368266
qRT-PCR: Atg4b-R	GTTCCCCCAATAGCTGGAAAG	
qRT-PCR: Atg5-F	TGTGCTTCGAGATGTGTGGTT	NM_053069.6
qRT-PCR: Atg5-R	TCAAATAGCTGACTCTTGG-CAA	
qRT-PCR: Atg7-F	GTTCGCCCCCTTTAATAGTGC	NM_001253717.2
qRT-PCR: Atg7-R	TGAACTCCAACGTCAAGCGG	
qRT-PCR: Atg9a-F	CAGTTTGACACTGAATAC-CAGCG	NM_001288612.2
qRT-PCR: Atg9a-R	AATGTGGTGCCAAGGTGATTT	
qRT-PCR: Atg16L1-1-F	CAGAGCAGCTACTAAGCGACT	NM_001205391.1
qRT-PCR: Atg16L1-1-R	AAAAGGGGAGATTCG-GACAGA	
qRT-PCR: Atg16L1-2-F	CAGAGCAGCTACTAAGCCACT	NM_029846.4
qRT-PCR: Atg16L1-2-R	ACAGAGCGTCCCCAAAGATA	
qRT-PCR: Atg16L1-3-F	AGCAGCTACGAGACGCTCT	NM_001205392.1
qRT-PCR: Atg16L1-3-F	CGCATCGAAGACATACGAGG	
qRT-PCR: Nrf1-F	AGCACGGAGTGACCCAAAC	NM_001361692.2
qRT-PCR: Nrf1-R	TGTACGTGGCTACATGGACCT	

qRT-PCR: Ppargc1a-F	TATGGAGTGACATAGAG-TGTGCT	NM_008904.3
qRT-PCR: Ppargc1a-R	CCAC-TTCAATCCACCCAGAAAG	
qRT-PCR: Ppargc1b-F	TCCTGTAAAAGCCCGGAGTAT	NM_133249.3
qRT-PCR: Ppargc1b-R	GCTCTGGTAGGGGCAGTGA	
qRT-PCR: Tfam-F	ATTCCGAAGTGTTTTTCCAGCA	NM_009360.4
qRT-PCR: Tfam-R	TCTGAAAGTTTTGCATCTGGGT	
qRT-PCR: Mnf1-F1	CCTACTGCTCCTTCTAACCCA	NM_026063.2
qRT-PCR: Mnf1-R1	AGGGACGCCAATCCTGTGA	
qRT-PCR: Mnf1-F2	AGAACTGGACCCGGTTACCA	NM_001364944.1
qRT-PCR: Mnf1-R2	CACTTCGCTGATACCCCTGA	
qRT-PCR: Dnm1L-F	TTACGGTTCCCTAAACTTCACG	NM_152816.4
qRT-PCR: Dnm1L-R	GTCACGGGCAACCTTTTACGA	
qRT-PCR: Opa1-F	CGACTTTGCCGAGGATAGCTT	NM_001403172.1
qRT-PCR: Opa1-R	CGTTGTGAACACACTGCTCTTG	
qRT-PCR: Pink1-F	GATGATGTGGAATATCTCGG-CAGGT	NM_026880.2
qRT-PCR: Pink1-R	CTTGGGACCATCTCTG-GATCTTCTG	
qRT-PCR: ULK1-F	AAGTTCGAGTTCTCTCGCAAG	NM_001347394.1
qRT-PCR: ULK1-R	CGATGTTTTCGTGCTTTAG-TTCC	
qRT-PCR: ULK2-F	AGCTTCAG-CATGAAAACATCGT	NM_013881.4
qRT-PCR: ULK2-R	CGATTGGCATAAGA-CAACAGGA	
qRT-PCR: Parkin2-F	TCTTCCAGTGTAACCACCGTC	NM_001317726.2
qRT-PCR: Parkin2-R	GGCAGGGAGTAGCCAAGTT	
qRT-PCR: Bnip3-F	GCTCCCAGACACCACAAGA-TACCAA	NM_009760.4
qRT-PCR: Bnip3-R	TGAGAG-TAGCTGTGCGCTTCGG	
qRT-PCR: Fundc1-F	CCCCCTCCCCAAGACTATGAA	NM_028058.4
qRT-PCR: Fundc1-R	CCACCCATTACAATCTGAG-TAGC	
qRT-PCR: Dapk1-F	ATGACTGTGTTCAGGCAGGAA	NM_029653.3
qRT-PCR: Dapk1-R	CCGGTACTTTTCTCAC-GACATTT	
qRT-PCR: Tfeb-F	CCACCCCAGCCATCAACAC	NM_001161722.1
qRT-PCR: Tfeb-R	CAGACAGATACTCCCGAAC-CTT	
qRT-PCR: SOD2-F	ACAACAGGCCTTATTCCGCT	NM_013671.3

qRT-PCR: SOD2-R	CCCCAGTCATAGTGCTGCAA	
qRT-PCR: CAT-F	CACTGACGAGATGGCACACT	NM_009804.2
qRT-PCR: CAT-R	TGTGGAGAATCGAACGGCAA	
qRT-PCR: GPX-F	CAGTCCACCGTGTATGCCTT	NM_008160.6
qRT-PCR: GPX-R	GTGTCCGAACTGATTGCACG	
qRT-PCR: MPO-F	CACATACCGGGACTACCTGC	NM_010824.2
qRT-PCR: MPO-R	GGGCCGGTACTGATTGTTCA	
qRT-PCR: NOX2-F	TCAGTGAGCTTTCCCTGTGTC	NM_007807.5
qRT-PCR: NOX2-R	ATTTGCCTTCGGTGATGTGC	
qRT-PCR: eNOS-F	GATGTGCTGCCCCTGTTACT	NM_008713.4
qRT-PCR: eNOS-R	ATGTTGGACACAGCTGGGAG	
qRT-PCR: DUOX1-F	AAAACGTCAGGGGAGCTCTG	NM_001099297.1
qRT-PCR: DUOX1-R	ACGCACTTTGGTCGAGGAAT	
qRT-PCR: Sdhaf2-F	AAAGAGGCAGTCACTCTGGC	NM_025333.4
qRT-PCR: Sdhaf2-R	TGGGAGCGTTGTGAGGTAAC	
qRT-PCR: NOS1-F	TTCAGCGCCTGTCCCTTTAG	NM_008712.3
qRT-PCR: NOS1-R	TTGATGAAGGACTCGGTGGC	
